# Elasticity-dependent response of malignant cells to viscous dissipation

**DOI:** 10.1007/s10237-020-01374-9

**Published:** 2020-08-12

**Authors:** Elisabeth E. Charrier, Katarzyna Pogoda, Robin Li, Rebecca G. Wells, Paul A. Janmey

**Affiliations:** 1 Institute for Medicine and Engineering and Center for Engineering MechanoBiology, University of Pennsylvania, Philadelphia, PA USA; 2 Division of Gastroenterology and Hepatology, Department of Medicine, Perelman School of Medicine, University of Pennsylvania, Philadelphia, PA USA; 3 Institute of Nuclear Physics Polish Academy of Sciences, 31342 Kraków, Poland; 4 Department of Physiology, University of Pennsylvania, Philadelphia, PA USA; 5 Center for Engineering Mechanobiology, University of Pennsylvania, Philadelphia, PA USA

**Keywords:** Viscoelasticity, Malignant cell, Mechanosensing, Viscosity sensing

## Abstract

The stiffness of the cellular environment controls malignant cell phenotype and proliferation. However, the effect of viscous dissipation on these parameters has not yet been investigated, in part due to the lack of in vitro cell substrates reproducing the mechanical properties of normal tissues and tumors. In this article, we use a newly reported viscoelastic polyacrylamide gel cell substrate, and we characterize the impact of viscous dissipation on three malignant cell lines: DU145 and PC3 derived from prostate and LN229 from brain. The spreading, motility and proliferation rates of these cells were analyzed on 1 kPa and 5 kPa elastic and viscoelastic gels. Surprisingly, the effect of substrate viscous dissipation on cell behavior depended on substrate stiffness for the three cell types tested. We conclude that viscoelasticity controls the spreading, proliferation and migration of malignant cells in vitro. These results highlight the critical role of viscous dissipation in the phenotype and proliferation of malignant cells, especially in stiff tumor environments.

## Introduction

Cell differentiation and proliferation are regulated by the biochemical and mechanical environment. Over the last two decades, numerous bio-inspired substrates for cell culture have been developed to reproduce the mechanical environment of biological tissues and understand how mechanical cues influence cell morphology and behavior. However, most of these bio-inspired materials are purely elastic soft hydrogels that do not resemble biological tissues, which are viscoelastic (Denisin and Pruitt 2016; Engler et al. 2006) and have shear loss moduli (*G*″) that are 10–20% of the storage moduli (*G*′) (Cheng et al. 2008; Pogoda et al. 2014; Mihai et al. 2015; Safshekan et al. 2017; Geerligs et al. 2008; Perepelyuk et al. 2016). The balance between *G*′ and *G*″ is disturbed during pathological processes such as cancer (Levental et al. 2009; Visscher et al. 2016; Omari et al. 2015) and fibrosis (Chen et al. 2013; Deffieux et al. 2015) where tissue stiffness increases while the ratio *G*″/*G*′ decreases (Perepelyuk et al. 2013).

Tumors and their microenvironments are often stiffer than surrounding normal tissues (Huang and Ingber 2005; Krouskop et al. 1998). This increased stiffness, quantified by an elastic modulus, promotes malignant cell proliferation (Paszek et al. 2005; Tilghman et al. 2010) and resistance to chemotherapy (Castells et al. 2012; Correia and Bissell 2012). Additionally, some tumors such as breast present a simultaneous increase in stiffness and loss of stress relaxation (Levental et al. 2010), whereas others such as gliomas do not appear to increase stiffness, but do increase viscous dissipation (Reiss-Zimmermann et al. 2015). Given the stimulatory effect of environmental mechanical properties on malignant cell proliferation and drug resistance (Pogoda et al. 2017; Zhang et al. 2017; Hui et al. 2017), it is critical to assess the effect of viscous dissipation on malignant cell phenotype to improve therapeutic strategies.

In this article, we use a new kind of soft viscoelastic polyacrylamide hydrogel (Charrier et al. 2018, 2020) that reproduces the mechanical properties of normal and tumor tissues. We used elastic and viscoelastic 1 kPa and 5 kPa gels to analyze the response of three malignant cell lines to substrate elasticity and viscosity and show that the cellular response to viscous dissipation is specific to each cell type and dependent on matrix stiffness.

## Results and discussion

### Viscoelastic gels with independently tunable elasticity and viscosity

**Fig. 1:**
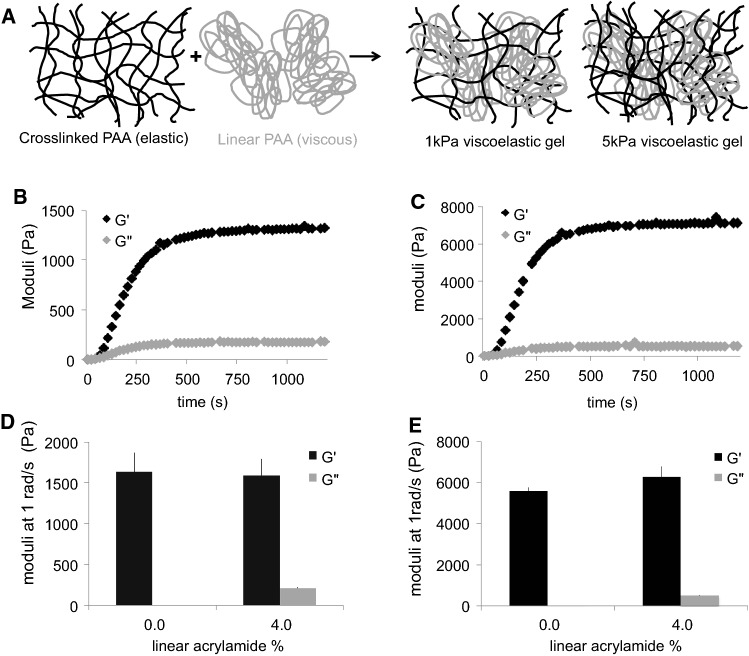
Mechanical characterization of elastic and viscoelastic gels. **a** Cartoon representing the organization of polyacrylamide in the viscoelastic gels. **b** Evolution of *G*′ and *G*″ over time during 1 kPa gel polymerization. **c** Evolution of *G*′ and *G*″ over time during 5 kPa gel polymerization. **d** Average *G*′ and *G*″ values for 1 kPa elastic and viscoelastic gels at 1 rad/s. n = 5 gels per condition. **e** Average *G*′ and *G*″ values for 5 k Pa elastic and viscoelastic gels at 1 rad/s. n = 5 gels per condition

Viscoelastic hydrogels are composed of linear polyacrylamide (PAA) molecules entangled in a cross-linked network of polyacrylamide (Fig. 1a). The formulation of the gels allows independent modification of their elasticity and viscosity. The elasticity is controlled by the amount of PAA cross-linked into a network; the viscosity is dependent on the amount of linear PAA sterically trapped inside this network (Table 1 and Fig. 1a). We formulated what we called 1 kPa and 5 kPa elastic and viscoelastic gels (Table 1) with loss (*G*″)/storage (*G*′) modulus ratios of 13% and 8%, respectively, when measured at 1 rad/s (Fig. 1d, e), which is similar to what is observed for biological tissues (Pogoda et al. 2014; Cheng et al. 2008; Mihai et al. 2015; Geerligs et al. 2008; Perepelyuk et al. 2016; Pogoda and Janmey 2018). *G*′ and *G*″ of the gels were measured with a rheometer during the polymerization of the network by applying an oscillating shear stress to the gels (Fig. 1b, c); characterization was at 1 rad/s because this timescale was previously reported as relevant for cell mechanosensing at the steady state (Plotnikov et al. 2012; Chan and Odde 2008).

**Table 1:** 1 kPa and 5 kPa elastic and viscoelastic gels average elastic (*G*′) and viscous (*G*″) moduli at 1 rad/s, and gel formulation

Gel	*G*′ (Pa)	*G*′′ (Pa)	% acrylamide	% bis-acrylamide	% linear PAA
1 kPa elastic	1600	1	4.5	0.1	0
1 kPa viscoelastic	1590	206	5.5	0.1	4
5 kPa elastic	5600	11	8	0.1	0
5 kPa viscoelastic	6300	490	8	0.15	4

The stiffness of the gel depends on the amount of acrylamide polymerized in the network (Fig. 1 and Table 1) such that gels containing about 5% acrylamide have an elastic modulus of ~ 1000 to 1500 Pa (marked 1 kPa) and gels containing 8% acrylamide have an elastic modulus of 5–6 kPa (marked 5 kPa). During the polymerization of the PAA network, both *G*′ and *G*″ increase over time (Fig. 1b, c). This result indicates that the loss moduli of the gels originate from the confinement of the linear PAA into a network. Additionally, the value of *G*″ is different for 1 kPa gels and 5 kPa gels, even though the amount of linear PAA in both gels is similar (Table 1). The value of *G*″ was correlated with that of *G*′, indicating that the linear PAA confers its dissipative properties to the gel, to an extent that depends on its mesh size and therefore elastic modulus. The network of PAA participates in viscous dissipation by controlling the movement of the linear polymers, thereby affecting their relaxation inside the gel. These gels have been described as viscoelastic solids that exhibit stress relaxation under the application of a constant shear strain (Charrier et al. 2018).

In order to enable cell adhesion to the gels, acrylamide was copolymerized with acrylic-acid N-hydroxy-succinimide ester prior to polymerization. The resulting network contained activated monomers within the polymer chain that were covalently cross-linked to collagen I after incubation with the adhesion protein at pH = 8.2 to prevent the formation of collagen I bundles and ensure that the gels would be coated with monomers of collagen I.

### Cell spreading and morphology on viscoelastic gels

We characterized the response to elasticity and viscous dissipation of three malignant epithelial cell lines from significantly different in vivo mechanical environments: the brain and bone. We used DU145 cells, which are prostate carcinoma cells derived from brain metastasis; PC3 cells, prostate carcinoma cells derived from bone metastasis; and LN229 cells, glioblastoma cells from primary tumors. By using these three cell types, we were able to test the response to viscosity of cells which in vivo had proliferated in a stiff and nearly purely elastic environment (PC3 cells), and those which had proliferated in a very soft and viscous environment (DU145 cells and LN229 cells).

**Fig. 2:**
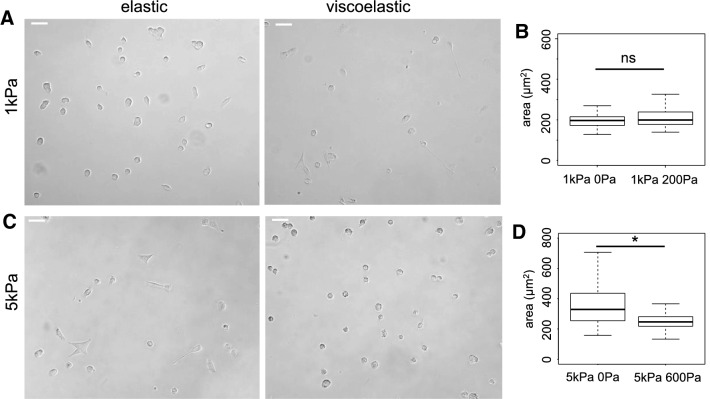
Morphology of DU145 prostate cells on 1 kPa elastic (*G*′ = 1 kPa; *G*″ = 0 Pa), viscoelastic (1 kPa; 200 Pa), 5 kPa elastic (5 kPa; 0 Pa) and viscoelastic (5 kPa; 600 Pa) gels. **a** Bright-field images of DU145 cells after 24 h on 1 kPa gels. Scale bar = 50 μm. **b** Average area of DU145 cells after 24 h on soft 1 kPa elastic and viscoelastic gels, *p < 0.001. **c** Bright field images of DU145 cells after 24 h on 5 kPa gels. Scale bar = 50 μm. **d** Average area of DU145 cells after 24 h on 5 kPa elastic and viscoelastic gels, *p < 0.001

**Fig. 3:**
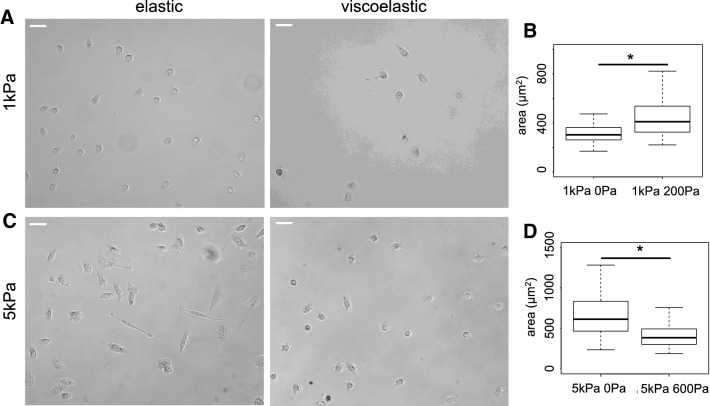
Morphology of PC3 cells on 1 kPa and 5 kPa elastic and viscoelastic gels. **a** Bright field images of PC3 cells after 24 h on 1 kPa PAA gels. Scale bar = 50 μm. **b** Average area of PC3 cells after 24 h on 1 kPa elastic and viscoelastic gels, *p < 0.001. **c** Bright field images of PC3 cells after 24 h on 5 kPa gels. Scale bar = 50 μm. **d** Average area of PC3 cells after 24 h on 5 kPa elastic and viscoelastic gels, *p < 0.001

**Fig. 4:**
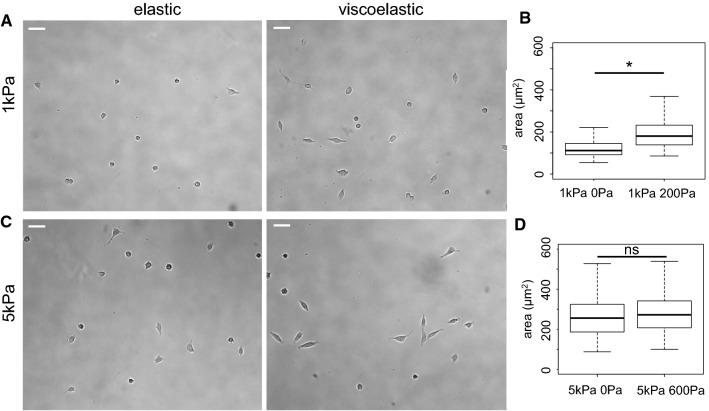
Morphology of LN229 cells on 1 kPa and 5 kPa elastic and viscoelastic gels. **a** Bright field images of LN229 cells after 24 h on 1 kPa PAA gels. Scale bar = 50 μm. **b** Average area of LN229 cells after 24 h on 1 kPa elastic and viscoelastic gels, *p < 0.001. **c** Bright field images of LN229 cells after 24 h on 5 kPa gels. Scale bar = 50 μm. **d** Average area of LN229 cells after 24 h on 5 kPa elastic and viscoelastic gels, *p < 0.001

We determined the projected area of the three cell types 24 h after plating on 1 kPa and 5 kPa elastic and viscoelastic gels (Figs. 2, 3 and 4). On purely elastic gels, DU145 cells were larger when *G*′ was 5 kPa than when it was 1 kPa, which demonstrates their ability to sense and respond to stiffness. On 1 kPa gels, DU145 cells had a similar area and round morphology, regardless of whether the gels were purely elastic (Circ = 0.90 ± 0.05) or viscoelastic (Circ = 0.91 ± 0.07) (Fig. 2b). On 5 kPa gels, however, DU145 cells were well spread and more elongated when the gels were elastic (Circ = 0.86 ± 0.15), but they remained round and small on viscoelastic gels (Circ = 0.93 ± 0.05) (Fig. 2d). On both 1 kPa and 5 kPa viscoelastic gels, the spreading area of DU145 cells was around 250 μm^2^, showing that, surprisingly, DU145 cell spreading is not affected by the elastic modulus of the substrate in a viscoelastic environment (Fig. 2b).

PC3 cells respond to elasticity similarly to DU145 cells, with increased cell spreading in response to increased elastic modulus of their substrate (Fig. 3) as previously shown (Kraning-Rush et al. 2012). The effect of viscous dissipation on PC3 cell spreading depends on the elastic modulus of the hydrogel. On 1 kPa gels, PC3 cells spread significantly more when gels were viscoelastic rather than purely elastic with slightly more elongated morphology (Circ = 0.88 ± 0.09 on viscoelastic and Circ = 0.93 ± 0.03 on elastic gels) (Fig. 3b). On 5 kPa gels, PC3 cell area was significantly smaller, and cells exhibited a rounder morphology on viscoelastic gels (Circ = 0.89 ± 0.07) than on elastic gels (Circ = 0.73 ± 0.21) (Fig. 3c). PC3 cells responded in opposite ways to viscous dissipation on soft 1 kPa and on stiffer 5 kPa gels.

Similar to the response of PC3 cells, LN229 cell area is larger on 5 kPa than on 1 kPa elastic gels (Fig. 4). This is consistent with previous observations that multiple glioma cell lines, including LN229, are substrate rigidity-sensitive and do not spread and elongate on soft hydrogels (Ulrich et al. 2009; Pogoda et al. 2017). LN229 cells respond differently to viscous dissipation as a function of substrate elasticity compared to DU145 and PC3 cells. LN229 cells spread significantly more and exhibit more elongated morphology (Circ = 0,61 ± 0,2) on 1 kPa viscoelastic gels than on 1 kPa elastic gels (Circ = 0,87 ± 0,1) (Fig. 4b), while they spread similarly on both types of 5 kPa gels with more elongated shape on viscoelastic (Circ = 0,47 ± 0,17) than elastic gels (Circ = 0,59 ± 0,22) (Fig. 4d).

For all cell lines studied, an increase in elastic modulus from 1 to 5 kPa was associated with an increase in spreading, as previously observed for other types of the cells (Mih et al. 2011; Engler et al. 2004; Bangasser et al. 2017; Pogoda et al. 2017). Many cell types present a biphasic response to elasticity (Bangasser et al. 2017), with an optimal spreading elasticity that is specific to each cell type (Kostic et al. 2009). On viscoelastic substrates, the cellular response to mechanical cues from the environment becomes more complex as energy dissipation through the underlying substrate occurs (Chaudhuri et al. 2015; Charrier et al. 2018). Our data suggest that the response of malignant cells to viscoelastic materials displays at least two regimes, depending on the elastic moduli of the substrates (Figs. 2, 3 and 4). At low elasticity, viscous dissipation tends to favor cell spreading by increasing the overall stiffness perceived by cells (Gong et al. 2018). At high stiffness, viscous dissipation tends to prevent spreading, presumably by dissipating a part of the cellular energy through focal adhesions and thus lowering the adhesion strength of cells to their substrate. The elasticity at which the effect of viscosity transitions from promoting to limiting cell spreading seems to be cell type specific. We conclude that cell spreading on a viscoelastic solid is controlled by an interplay between the elastic and the viscous moduli of the substrate.

### Cell motility on elastic and viscoelastic gels

In vivo*,* malignant cells from tumors can migrate through tissues to establish secondary tumors; as a consequence, the migratory patterns of malignant cells are an important part of their phenotype. The mechanical environment of malignant cells regulates their locomotion, and an increase in stiffness often, but not always, correlates with increased motility (Pathak and Kumar 2012; Pogoda et al. 2017; Ulrich et al. 2009).

**Fig. 5:**
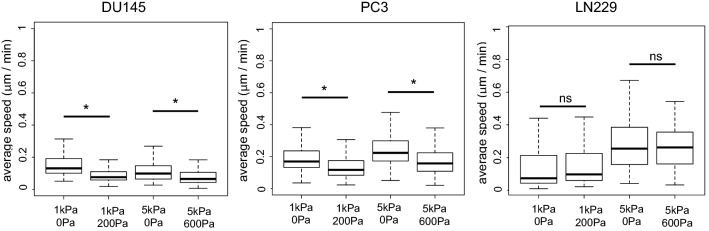
Average motility speed of the three types of malignant cells on soft elastic and viscoelastic hydrogels

We observed that PC3 and LN229 cell migration speed was sensitive to elastic modulus. These cell types were faster on 5 kPa than on 1 kPa elastic gels (Fig. 5). However, DU145 cells had similar speed on 1 kPa and 5 kPa elastic gels. We then analyzed the effect of viscous dissipation on cell motility. The two prostate cell lines (DU145 and PC3) responded to viscosity in a similar way, showing decreased motility on viscoelastic substrates. The glioma cells (LN229) had similar motility on elastic and viscoelastic gels of the same elastic modulus.

As previously shown, viscoelasticity impaired the formation of mature focal adhesions (Cameron et al. 2011; Charrier et al. 2018) and thus might inhibit cell migration by preventing the establishment of the stable contacts with the substrate required to push the cell forward (Nagano et al. 2012). Moreover, the contractile forces produced by the actomyosin system regulate cell migration. Dissipating part of this contractile energy through the substrate might decrease the amount of force available to move the cell and thus might slow down migration similar to the effect of actomyosin inhibitors (Ivkovic et al. 2012).

Migration and spreading have been reported to be correlated on elastic substrates (Tilghman et al. 2010) through the control of focal adhesion (FA) size (Kim and Wirtz 2013). Cells on viscoelastic substrates form smaller FAs than on elastic substrates of the same elasticity (Charrier et al. 2018; Cameron et al. 2011). The formation of FAs and therefore the rate and extent of spreading depend on the relative rates or engagement of the adhesion complexes, motors, an adapter proteins (clutches) for the FA. A recent model that integrates viscous and elastic responses of the substrate with the on and off rates of molecular clutches predicts either increased or decreased spreading of cells in viscoelastic compared to elastic substrates, depending on the relative magnitudes of the relaxation times of the substrate and the proteins within the FA. Since the complement of molecular clutches including talin, filamin, vinculin and other proteins is unique to each cell type and might change as the stiffness or viscosity of the substrate changes, it is reasonable that different cell types can have opposite responses to viscosity depending on the magnitude of the elastic modulus and the nature of the adhesion receptors. There are insufficient quantitative data for the kinetic constants and amounts of FA proteins in different cell types, so direct application of this model to compare different cell types is not yet feasible. However, it is important to highlight that these mechanisms exhibit strong cell-type dependency, as the specific characteristics of a cell type will define how it interacts with its substrate (Webb et al. 2000).

### Cell proliferation on elastic and viscoelastic gels

**Fig. 6:**
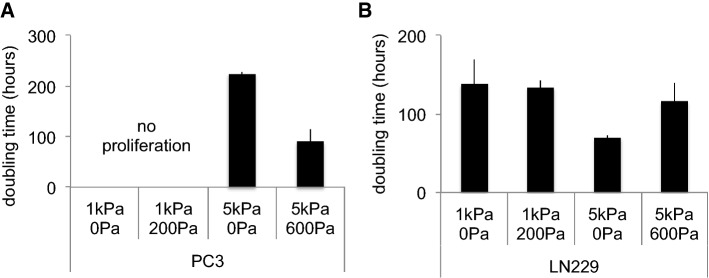
Average doubling time of PC3 (A) and LN229 (B) cells cultivated on elastic or viscoelastic soft PAA hydrogels

Stiff matrices often favor cell proliferation while soft substrates trigger growth-arrest (Klein et al. 2009; Tilghman et al. 2010; Ulrich et al. 2009; Mih et al. 2011). If malignant cell growth on hydrogels correlates with the rate of tumor growth in vivo (Tilghman et al. 2010), we speculated that the viscosity of soft tissues could also affect the cell proliferation rate in vivo because tumor tissues are stiffer than the surrounding normal tissue and characterized by altered viscous dissipation (Levental et al. 2010). We therefore tested whether viscoelasticity affects tumor cell proliferation in vitro. The three cell lines were cultivated on elastic and viscoelastic gels for 4 days, and the average doubling time of the cell population was quantified (Fig. 6).

We were not able to study the proliferation of DU145 cells as their number did not increase over time on hydrogels in any condition tested. This was unexpected, as these cells proliferate in vivo to establish secondary tumors in the very soft environment of the brain. However, on hydrogel substrates, the cells are able to adhere only by receptors to the single protein, collagen in this case, covalently linked to the gel surface; in vivo as well as on glass or plastic, there are likely to be multiple types of surface-bound receptors required to drive proliferation. Alternatively, since soft substrates have been reported to promote apoptosis (Leight et al. 2012; Tilghman et al. 2010), the lack of proliferation of DU145 cells might also be partially due to the programmed death of part of the population.

No proliferation was observed for PC3 cells on 1 kPa gels, and we hypothesize that 1 kPa is too soft to activate their proliferation. However, PC3 cells were able to proliferate on 5 kPa gels and interestingly they proliferated faster on viscoelastic gels than on elastic gels. PC3 cell growth was previously described as rigidity-independent (Tilghman et al. 2010) but our results demonstrate that PC3 cells respond to mechanical changes affecting the viscoelasticity of the matrix. We hypothesize that the elastic and the viscous moduli are sensed through different cellular pathways.

LN229 cells proliferated on all the substrates tested. Glioblastoma cells have been reported to grow faster on stiff substrates (Pogoda et al. 2017; Ulrich et al. 2009). Our results are in agreement with these observations as LN229 cells proliferate faster on 5 kPa elastic gels than on 1 kPa elastic gels. LN229 growth was comparable on 1 kPa elastic and viscoelastic gels, while on 5 kPa gels cell proliferation was significantly slowed by viscous dissipation.

In summary, proliferation analyses of the three cell lines showed that the effect of viscous dissipation is different for each cell type and not simply dependent on matrix elasticity. This observation was surprising given that population doubling time has been previously reported to correlate with cell spreading and adhesion (Mih et al. 2012; Ulrich et al. 2009; Pogoda et al. 2017). However, our data suggest that viscous dissipation could be a powerful regulator of malignant cell phenotype and proliferation, especially in tumor environments presenting alterations of both elastic and viscous moduli (Levental et al. 2010).

## Conclusions

We describe here the use of a newly developed viscoelastic hydrogel, which reproduces the viscoelasticity of biological tissues and is suitable for the culture of multiple cell types. By cultivating malignant cells of different origins on this material, we showed that the impact of viscous dissipation on cell phenotype depends on both substrate elastic modulus and cell type. All three types of cancer cells PC3, DU145 and LN229 respond to viscoelasticity of the substrate at least at two regimes, depending on the elastic moduli *G*′ of the substrates in terms of morphology, motility and proliferation (different viscosity sensing at 1 kPa and 5 kPa elasticity, Table 2). Moreover, viscous dissipation modulates the cellular response to elasticity in a cell type-dependent manner; the response is more complex than cells sensing viscoelastic substrates as softer. This innovative material has potential to reveal the role of viscous dissipation in pathological contexts such as tumor progression.

**Table 2:** Summary of the stiffness-dependent viscosity sensing by the PC3, Du145 and LN229 cells in terms of their ability to spread (morphology), migrate (motility) and grow (proliferation)

	Viscosity sensing at 1 kPa stiffness	Viscosity sensing at 5 kPa stiffness	
PC3	Yes	Yes	Morphology
DU145	No	Yes	
LN229	Yes	No	
PC3	Yes	Yes	Motility
DU145	Yes	Yes	
LN229	No	No	
PC3	–	Yes	Proliferation
DU145	–	–	
LN229	No	Yes	

–Depicts the conditions were no proliferation was observed

## Methods

### Polyacrylamide gels

Viscoelastic PAA gels are made in two steps. First, the linear PAA is polymerized. Second, the network of PAA is polymerized around these linear molecules of PAA.

The linear solution is obtained by adding 0.05% TEMED and 0.024% ammonium persulfate to a PAA 5% (w/v) acrylamide aqueous solution which was previously degassed and polymerized overnight at 37 °C. The resulting viscous fluid contains long linear PAA molecules and can be stored at 4 °C protected from light.

To obtain the network of PAA, 300 μL of linear PAA solution was mixed with acrylamide, bis-acrylamide and H_2_O at a final volume of 500μL. Polymerization was triggered by adding 1.25 μL TEMED and 3.75 μL 10% ammonium persulfate to the 500 μL of gel mix. The gel was polymerized for 30 min at room temperature and rinsed three times with PBS. Table 1 describes the formulation of the 1 kPa and 5 kPa gels.

### Rheology

Rheology experiments were performed with a Kinexus stress-controlled rheometer (Malvern) and an RFS3 strain-controlled rheometer (TA Instruments) with a 20 mm circular parallel-plate geometry. The gels were polymerized for 30 min between the plates of the rheometer before starting the measurements. The evolution of *G*′ and *G*″ during polymerization was followed by applying a 2% shear strain at the frequency of 1 rad/s until the values of *G*′ and *G*″ reached a plateau.

### Gel functionalization

To cross-link collagen I to the gel, 50 μL of 4% acrylic-acid N-hydroxy-succinimide ester (NHS) in DMSO was added to 500 μL of unpolymerized acrylamide and bis-acrylamide gel solution. NHS coupled to the acrylate monomers, becomes reactive at high pH and can covalently bind proteins such as collagen I. This method ensured specific activation of the network of PAA as only monomers within the network are activated. The linear PAA previously polymerized is inert and thus will not present collagen I. Gels were functionalized with a solution of rat tail collagen I (Corning) at 0.1 mg/mL in 50 mM HEPES buffer at pH = 8.2. The pH was carefully controlled to ensure that collagen I monomers were cross-linked to the network of PAA.

### Cell culture

DU145, PC3 and LN229 cell lines were purchased from the ATCC and were maintained in RPMI (Gibco) medium (DU145 and PC3) or DMEM (Sigma) medium (LN229) supplemented with 10% fetal bovine serum (ATCC) and 1% of penicillin/streptomycin (Gibco) at 37 °C and 5% CO_2_. For experiments, cells were plated at a density of 15,000 cells/cm^2^ on PAA hydrogels in a volume of 100 μL to ensure that the cells were in contact with the gel.

### Cell morphology analysis

After 24 h of cell growth, bright-field images were taken with a Leica DMIRE2 inverted microscope (Leica) equipped with a Hamamatsu camera, and single-cell areas were manually traced using ImageJ software. Cell circularity was determined using the formula $\mathrm{Circ}=4\pi (\frac{\mathrm{area}}{{\mathrm{perimeter}}^{2}})$. Circularity value of 1.0 indicates a perfect circle. Approximately 100 cells per condition were analyzed.

### Cell migration experiment

The migration speed of cells growing on gels was determined with time-lapse microscopy over a period of 4 h. A Tokai-Hit Imaging Chamber (Tokai Hit, Shizuoka-ken, Japan) that maintained a humid 37 °C and 5% CO_2_ environment was first equilibrated for 1 h. After 24 h of seeding, cultures were placed inside the chamber mounted on a Leica DMIRE2 inverted microscope equipped with an ASI *x*/*y*/*z* stage (BioVision Technologies) and a Hamamatsu camera; a 10 × air lens was used for image sequence recording. Cell migration speed υ (length of the total trajectory *d* divided by time *t*) was calculated by tracing the (*x*, *y*) position of the center of the cell at every image using ImageJ Software (NIH) and the Manual Tracking plug-in (https://imagej.nih.gov/ij/). Minimum 70 cells per condition were analyzed.

### Cell proliferation experiment

Cells were plated on gels for 24 h, and then low-magnification bright-field images were taken. After 48 h and 72 h, images were taken again at the exact same positions. The number of cells was counted for each time point. The evolution of the number of cells over three days was then fitted with an exponential model: *y*(*t*) = *y*_0_e^*kt*^, where *y*_0_ is the cell number at the time point *t*_0_, *k* is the growth constant and *t* is time. Population doubling time (*t*_d_) was calculated using the equation ${t}_{\mathrm{d}}=\frac{\mathrm{ln}2}{k}$, as previously described (Pogoda et al. 2017).

### Statistical analysis

Each experiment was performed at least in triplicate. Nonparametric multiple comparisons for relative contrast effects test (R software package) using Tukey’s method with 95% confidence interval were used to confirm statistical differences between the measured quantities. Denotations: *, *P* ≤ 0.05; **, *P* ≤ 0.01; ***, *P* ≤ 0.001; ns, *P* > 0.05, no significant differences.
